# Medical Progress and Medical Journals

**Published:** 1877-01-20

**Authors:** Robert C. Word


### SOUTHERN MEDICAL RECORD.

VOL. VII. ATLANTA, GA., JANUARY 20, 1877. No. 1.

THOMAS S. POWELL, M. D., EDITOR.

W. T. GOLDSMITH, M. D., Assistant Editor.

R. C. WORD, M. D., Assistant Editor and Business Manager.

SUBSCRIPTION: TWO DOLLARS PER ANNUM, IN ADVANCE.

☞ All communications, and letters on business connected with THE RECORD for 1877 must be addressed to the Business Manager.

### Original and Selected Articles.

### MEDICAL PROGRESS AND MEDICAL JOURNALS.

BY ROBERT C. WORD, M.D.

Under the investigations of modern science, old theories are being exploded, important discoveries made, and new developments constantly occuring in every department of human knowledge.

It may truthfully be said that in no other field has there been greater and more rapid progress than in medicine, and the branches collateral to it.

The discoveries in chemistry, in the last twenty years, constitute it almost a new science. Physiology has made rapid strides, unfolding to our comprehension mysteries hitherto unknown in regard to the processes of nutrition, secretion, calorification, etc. The microscope has contributed largely to these discoveries, and given us further insight into the development and the growth of tissues. The advances in gynecology, the use of anæsthetic agents, and the improved methods of diagnosis have been many and important.

New appliances in surgery, and new agents in therapeutics, are so numerous and valuable that the physician who neglects to read the medical journals will soon find himself distanced by his competitors, and numbered among the ignorant and non-progressive

A short time ago the writer was called to consult with a practitioner at a distant country location, who, though a man of some intelligence, had neglected to take and to read a medical journal since the commencement of the Confederate war. His patient was suffering intense pain, and had been vomiting until exhaustion was alarming, and death appeared iminent. As the stomach rejected every remedy, a third of a grain of morphine was hypodermically injected. The result was a speedy and complete relief to all the symptoms. The attending physician expressed great astonishment, having never seen or heard of a hypodermic syringe. We opine if this man were to peruse the late volumes of THE SOUTHERN MEDICAL RECORD he would find himself very much in the condition of old Rip Van Winkle when first he beheld the strange faces around him, and gazed at the changes which had been wrought during his twenty years' nap.

The instance to which we have referred shows the importance of medical journals to the practicing physician. They are indispensible. Indeed, it is due to his own conscience and self-respect, and is justly expected by his patrons, that the man who assumes to treat the sick should keep pace with the progress of his art, and be prepared to give his patients the benefit of the latest and most approved agencies known to the medical world. To do this it is absolutely necessary that he take one or more good medical journals. If he has the means, it were well to take two; the one a quarterly, wherein subjects are treated exhaustively; the other a monthly or weekly, wherein he may find and promptly avail himself of all that is new and practical in the field of medical progress. The former may be dispensed with by the occasional purchase of standard new books; but the latter the busy practitioner can not dispense with without greatly impairing his success and disregarding his conscientious obligations to his profession and his patients.

For a thing so indispensible and important, it will not do to plead the cost as an excuse. A single visit or office prescription will offset the price of subscription, while often a single item in the ournal will repay the amount a hundred fold, to say nothing of the satisfaction and pleasure it affords to read the medical news, and to consult the views and experience of medical writers, in all parts of the world, upon the varied and interesting subjects connected with the profession.

